# Harzianopyridone Supplementation Reduced Chromium Uptake and Enhanced Activity of Antioxidant Enzymes in *Vigna radiata* Seedlings Exposed to Chromium Toxicity

**DOI:** 10.3389/fpls.2022.881561

**Published:** 2022-07-04

**Authors:** Anis Ali Shah, Adnan Noor Shah, Muhammad Bilal Tahir, Asad Abbas, Sumera Javad, Sajid Ali, Muhammad Rizwan, Saqer S. Alotaibi, Hazem M. Kalaji, Arkadiusz Telesinski, Talha Javed, Hamada AbdElgawad

**Affiliations:** ^1^Department of Botany, Division of Science and Technology, University of Education, Lahore, Pakistan; ^2^Department of Agricultural Engineering, Khwaja Fareed University of Engineering and Information Technology, Rahim Yar Khan, Pakistan; ^3^Department of Physics, Khwaja Fareed University of Engineering and Information Technology, Rahim Yar Khan, Pakistan; ^4^School of Horticulture, Anhui Agricultural University, Hefei, China; ^5^Department of Botany, Lahore College for Women University, Lahore, Pakistan; ^6^Department of Horticulture, Faculty of Agricultural Sciences and Technology, Bahauddin Zakariya University, Multan, Pakistan; ^7^Department of Environmental Science and Engineering, Government College University, Faisalabad, Pakistan; ^8^Department of Biotechnology, College of Science, Taif University, Taif, Saudi Arabia; ^9^Department of Plant Physiology, Institute of Biology, Warsaw University of Life Sciences, Szkoła Główna Gospodarstwa Wiejskiego (SGGW), Warsaw, Poland; ^10^Institute of Technology and Life Sciences-National Research Institute, Falenty, Poland; ^11^Department of Bioengineering, West Pomeranian, University of Technology in Szczecin, Szczecin, Poland; ^12^College of Agriculture, Fijian Agriculture and Forestry University, Fuzhou, China; ^13^Department of Botany and Microbiology, Faculty of Science, Beni-Suef University, Beni Suef, Egypt

**Keywords:** mung bean, chromium, stress, growth, harzianopyridone

## Abstract

This study explains the scarce information on the role of harzianopyridone (HZRP) in the alleviation of chromium (Cr) stress alleviation in *Vigna radiata* (L.). To this end, *V. radiata* seedlings primed with HZRP at 1 and 2 ppm were exposed to 50 mg kg^–1^ Cr for 30 days. Cr stress reduced growth, chlorophyll (Chl) content, net photosynthetic rate, gas-exchange attributes along with enhanced oxidative damages, i.e., electrolyte leakage (EL), hydrogen peroxide (H_2_O_2_), and malondialdehyde (MDA). Application of HZRP enhanced intercellular carbon dioxide (CO_2_) concentration, stomatal conductance, and net photosynthetic rate with decreased activity of the chlorophyllase (Chlase) enzyme in *V. radiata* seedlings exposed to Cr stressed conditions. To maintain Cr-induced oxidative damages, HZRP treatment increased the levels of antioxidant metabolites (phenolic and flavonoids) and the activity of antioxidative enzymes [superoxide dismutase (SOD), catalase (CAT), and peroxidase (POD)] in *V. radiata* seedlings grown in normal and Cr-polluted potted soil. In addition to this, glycine betaine content was also increased in plants grown in Cr-contaminated soil. It is proposed the potential role of supplementation of HZRP in mitigating Cr stress. Further research should be conducted to evaluate the potential of HZRP in the mitigation of abiotic stresses in plants.

## Introduction

Excessive deposition of heavy metal toxicants in agricultural soils leads to growth retardation and hindrance of normal physiological processes in plants. Heavy metals accumulation in edible parts of plants poses various damages to animals and human health (Ihtisham et al., 2021; Zaynab et al., 2022). Chromium (Cr) is the 21st most abundant heavy metal and is regarded as one of the most potent pollutants in the environment. Cr is a transition metal and exists in two common oxidation states, i.e., hexavalent Cr (Cr^6+^) and trivalent Cr (Cr^3+^) (Singh et al., 2021). Cr^6+^ is more toxic, mobile, and exerts carcinogenic effects on living organisms (Bharagava and Mishra, 2018). Cr^6+^ uptake in plants is facilitated through sulfate and phosphate pathways and is easily transported to various parts of plants (Devi and Kumar, 2020). Chromates and dichromates are hexavalent Cr compounds and are mostly used in Cr stress tolerance mechanisms (Shah et al., 2020).

The accumulation of increased Cr content in soil and subsequently in different portions of plants affects plants and human health (Sharma et al., 2020). Moreover, increased Cr concentration in the soil affects the growth, photosynthesis, metabolism, biomass production, and yield of several crops (Anjum et al., 2017; Singh P. et al., 2020). For instance, higher Cr levels disturb physiological and biochemical changes in plants, leading to reduced yield and productivity (Anjum et al., 2017; Singh P. et al., 2020). In addition, the growth of mung beans is severely hampered by Cr stressed conditions (Jabeen et al., 2016; Husain et al., 2021). Cr^6+^ toxicity disturbs homeostasis in plants due to the enhanced accumulation of reactive oxygen species (ROS). Increased accumulation of Cr results in oxidative damage in plant tissues through enhanced production of hydrogen peroxide (H_2_O_2_), malondialdehyde (MDA) content, electrolyte leakage (EL), and ROS levels (Singh P. et al., 2020). ROS production causes oxidative stress in plants and results in oxidative modification of nucleic acids, proteins, and lipids (Huang et al., 2019). At the cellular level, Cr accumulation results in increased ROS production, as proved by an enhanced level of MDA, EL, and H_2_O_2_ in plants exposed to Cr toxicity (Yu et al., 2018; Patra et al., 2019). Cr (VI) toxicity causes more damage as compared with Cr (III) toxicity in plants (Beyersmann and Hartwig, 2008). Cr (VI) accumulation disturbs ROS homeostasis in plants grown in Cr polluted soil (Maqbool et al., 2018; Zaheer et al., 2019; Tirry et al., 2021). The accumulation of ROS enhances lipid peroxidation besides the oxidation of crucial biomolecules (Wakeel et al., 2019; Askari et al., 2021). ROS accumulation changes the morpho-physiology and architecture of plants facing Cr stressed conditions (Sharma et al., 2022).

To mitigate heavy metal-induced oxidative stress, plants regulate the activities of various enzymatic and non-enzymatic antioxidants. In the case of severe heavy metal toxicity, plant metabolomics is negatively affected resulting in disruption of some biomolecules, which leads to oxidative stress (Paithankar et al., 2021; Sarraf et al., 2022). However, Cr toxicity in plants depends on the concentration of Cr uptake from the rhizospheric region (Wakeel et al., 2018, 2019; Farid et al., 2019). This results in disturbed nutrient translocation in plants due to Cr binding with membranous H^+^-ATPase and other carrier channels (Shahid et al., 2017). Antioxidant enzymes in plants reverse the deleterious effect of ROS produced by various mechanisms. Crucial antioxidant enzymes in plants include CAT, POD, and SOD (Zaheer et al., 2020). Cr stress affects the activity of antioxidant enzymes in plants (Zaheer et al., 2022).

*Vigna radiata* is a short-duration legume crop, cultivated predominately in Asia and other regions of the world (Nair et al., 2019). *V. radiata* is rich in nutritional content such as proteins, vitamins, dietary fibers, minerals, and a huge number of bioactive compounds (Hou et al., 2019).

Harzianopyridone (HZRP) is a *Trichoderma harzianum* secondary metabolite containing a penta-substituted pyridine ring system with a 2,3-dimethoxy-4-pyridinyl pattern. It is a volatile organic compound that has been reported to have active defensive mechanisms in plants and regulates growth in tomato, canola, and pea plants (Vinale et al., 2013; Stewart and Hill, 2014). In the study of Hermosa et al. (2012), HZRP may promote plant growth *via* auxin-like activity at low doses, but confer an antimicrobial effect at higher concentrations. Despite the utilization of HZRP as a promising metabolite to promote plant growth, its potential role in improving plant growth under heavy metal toxicity, e.g., Cr, is not yet evaluated. Thus, the current research was conducted to test the potential of HZRP in the alleviation of Cr toxicity and regulation of growth in *V. radiata* seedlings. To our knowledge, this study exploits the effect of HZRP on the growth and morpho-physiological characteristics of *V. radiata*.

## Materials and Methods

The experiment was conducted in the wirehouse of the Department of Botany, University of Education. A *V. radiata* cultivar, Inqalab Mung, was used during the experiment. Seeds of *V. radiata* were surface sterilized with 0.01% mercuric chloride for 5 min, followed by washing with double-distilled H_2_O. For Cr toxification, K_2_Cr_2_O_7_ was used during the experiment. Then, 50 mg kg^–1^ was added to the potting soil. This toxic Cr concentration refers to agricultural contaminated sites near District Lahore, Pakistan. Agricultural contaminated sites were irrigated with toxic effluents from the Hudiara drain. In the case of control (C) treatment, only distilled H_2_O was added to the soil. HZRP was purchased from Sigma Aldrich. Two concentrations of HZRP were used during the experiment, i.e., 1.0 and 2.0 ppm. Seeds of *V. radiata* were primed in HZRP solutions for 2 h. A completely randomized design (CRD) was used during the experiment. After 3 weeks, the root and shoot length of *V. radiata* were determined.

### Assessment of Photosynthetic Pigments

Photosynthesis pigment and other photosynthetic factors were determined. The chlorophyll (Chl) content of leaves was evaluated in a non-destructive manner throughout the experiment with a Chl meter, SPAD 402 PLUS (Minolta, Japan). Using an infrared gas analyzer (LI-6400XT, Portable Photosynthesis System, LI-COR, NE, United States), the net photosynthetic rate, intercellular carbon dioxide (CO_2_) concentration, and stomatal conductance of the topmost fully developed leaves of *V. radiata* plants were recorded.

### Determination of Reactive Oxygen Species Content

The methodology described by Velikova et al. (2000) was used to determine the H_2_O_2_ content in the leaves of *V. radiata* plants. Supernatant (0.5 ml) was mixed with 0.5 ml of 10 mM phosphate buffer (pH 7.0) having 1 ml potassium iodide following extraction in 5 ml of 1 M TCA (0.1 w/v) to determine H_2_O_2_. Using the extinction coefficient of 0.28 M1 cm1 and the content expressed as nmol g1 fresh weight (FW), the content of H_2_O_2_ was determined after taking the absorbance at 390 nm (FW).

### Plant Extract and Enzyme Activity Assay

Fresh leaf samples from *V. radiata* plants were collected and mashed at 4°C in a pre-chilled pestle mortar for the enzyme assays. The 0.5-g powder was added to three volumes (w/v) of cold extraction medium buffer, which contained potassium phosphate buffer (100 mM) pH 7.0, 0.5% Triton X-100, and 1% polyvinylpyrrolidone. Following 20-min centrifugation at 15,000 g at 4°C, the supernatants were utilized in the experiments afterward. The extraction buffer was spiked with 2 mM ascorbate to estimate ascorbate peroxidase (APX). Filtered homogenates were centrifuged for 20 min at 4°C at 15,000 g. Protein and enzyme activity tests were done with the supernatants.

The activity of the enzyme chlorophyllase (Chlase) was evaluated according to the methods of McFeeters et al. (1971) and Fang et al. (1998). Using the method described by Jain and Gadre (2004), the activity of aminolevulinic acid dehydratase (ALAD) in *V. radiata* leaves was measured by quantifying the production of porphobilinogen (PBG) spectrophotometric value at 553 nm for 15 min against a zero-time control. Using this definition, one unit of enzyme activity was defined as 1 nmol of PBG produced per hour per gram (h^–1^g^–1^) of freshly harvested leaf weight (LW).

Spectrophotometric analysis at 560 nm was used to calculate the activity of SOD (Giannopolitis and Ries, 1977). The activity of APX was determined using the Nakano and Asada (1981) procedure.

### Determination of Total Phenolic Content and Flavonoids

The Folin-Ciocalteau method was used for the estimation of total phenolic content (Ordon et al., 2006). Then, a 0.5-ml plant sample was mixed with Folin-Ciocalteau reagent (0.2 N) for 5 min and sodium carbonate (2.0 ml of 75 g/L). After 2 h, the absorbance of the reaction was carried out at 760 nm at room temperature.

The method of Sarker and Oba (2018) was used for the determination of flavonoid content in the leaf extract. Leaf extract (500 μl), methanol (1.5 ml), potassium iodide (1 M), and aluminum chloride (1 ml of 10%) were allowed to stand for half an hour in the test tube at room temperature. Absorbance was calibrated at 415 nm using a spectrophotometer (Hitachi, Tokyo, Japan).

### Determination of Chromium Content

Root and shoot samples of *V. radiata* seedlings were dried in an oven and digested with the help of HCLO_4_. The Cr content in the study samples was determined with the help of an atomic absorption spectrophotometer (SpectraAA-220FS).

### Statistical Analysis

The SPSS software version 20.0 was applied for the analysis of the variance of the obtained data. The mean values obtained during the research were compared by employing Duncan’s multiple range test (DMRT) at *p* ≤ 0.05. The data depicted are mean ± *SE*, where *n* = 5.

## Results

### Harzianopyridone Increased Growth of *Vigna radiata* Grown Under Control and Chromium Stress Conditions

Table 1 shows that Cr stress reduced root and shoot length by 57 and 38%, respectively, as compared with C-treated *V. radiata* seedlings. In contrast, HZRP priming increased the root, and shoot length of treated seedlings in normal as well as Cr-polluted soil. In the case of soil spiked with Cr, HZRP, mainly at a concentration of 2 ppm, enhanced root and shoot length by 85 and 33% as compared to Cr-only treatment.

**TABLE 1:** Effect of harzianopyridone (HZRP) on root and shoot length of *Vigna radiata* exposed to chromium (Cr) stress.

Treatments	Root length (cm)	Shoot length (cm)
C	11 ± 0.89bc	29 ± 1.76bc
Cr	7 ± 0.54d	21 ± 1.05d
HZRP1	14 ± 1.03ab	33 ± 2.18b
HZRP2	15 ± 0.72a	38 ± 2.89a
Cr + HZRP1	10 ± 0.38c	25 ± 1.27cd
Cr + HZRP2	13 ± 1.28b	28 ± 1.47c

*Different letters indicate significant differences among the treatments (p ≤ 0.05). Cr, 50 mg kg^–1^, HZRP1, 1 ppm; HZRP2, 2 ppm.*

### Harzianopyridone Reduced Chromium Accumulation in the Content in Root and Shoot of *Vigna radiata*

Harzianopyridone priming reduced Cr uptake in *V. radiata* seedlings exposed to Cr stress. A high Cr value was found in the roots of *V. radiata* seedlings exposed to Cr-alone treatment (Table 2). Priming with HZRP at 2 ppm reduced Cr content in the root and shoot of *V. radiata* seedlings by 80.7 and 78.9%, respectively, in comparison with Cr-treatment.

**TABLE 2:** Effect of HZRP on Cr content in the root and shoot of *V. radiata* exposed to Cr stress.

Treatments	Cr content in root (μg g^–1^ DW)	Cr content in shoot (μg g^–1^ DW)
C	ND	ND
Cr	1.97 ± 0.056a	0.34 ± 0.034a
HZRP1	ND	ND
HZRP2	ND	ND
Cr + HZRP1	1.17 ± 0.029b	0.21 ± 0.073ab
Cr + HZRP2	1.09 ± 0.062bc	0.19 ± 0.081b

*Different letters indicate significant differences among the treatments (p ≤ 0.05). Cr, 50 mg kg^–1^, HZRP1, 1 ppm; HZRP2, 2 ppm.*

### Harzianopyridone Improved Chlorophyll Metabolism and Photosynthetic Reactions in *Vigna radiata*

**FIGURE 1:**
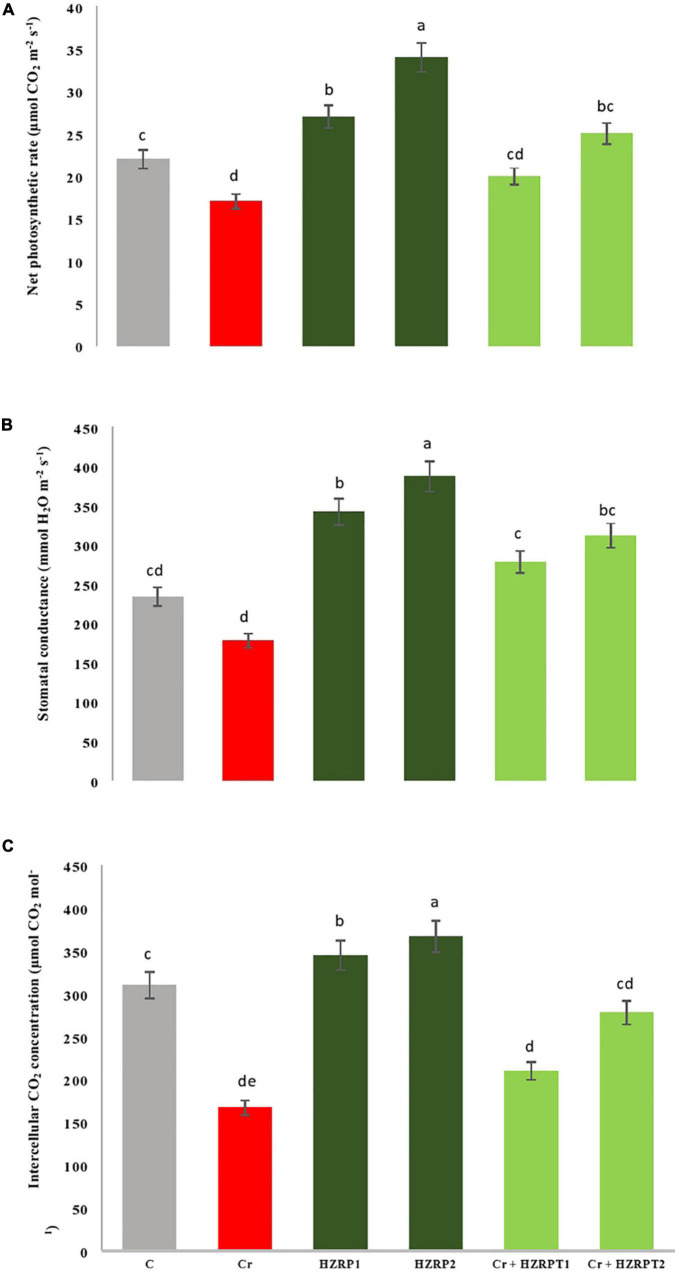
Effect of harzianopyridone on net photosynthetic rate **(A)**, stomatal conductance **(B)** and intercellular CO_2_ concentration **(C)** in *V. radiata* seedlings grown in Cr toxificated soil. Different letters indicate significant difference among the treatments (*p* < 0.05). Cr, 50 mg kg^–1^, HZRP1, 1 p.p.m; HZRP2, 2 p.p.m.

Chromium stress reduced the net photosynthetic rate in *V. radiata* seedlings by 29% as compared with the control treatment. On the other hand, HZRP supplementation increased the net photosynthetic rate in *V. radiata* seedlings grown in normal and Cr-toxificated soil. In contrast, 2 ppm of HZRP increased the net photosynthetic rate by 47.05% in *V. radiata* seedlings grown in Cr-toxic soil, in comparison with Cr-only treatment. To understand the bases of increased photosynthesis, stomatal, and non-stomatal parameters were measured. At the stomatal level, HZRP at 2 ppm significantly enhanced stomatal conductance and intercellular CO_2_ concentration in *V. radiata* seedlings grown in normal and Cr-toxificated soil, as compared with the Cr-only treatment (Figure 1). Regarding the non-stomatal parameters, Cr toxicity reduced Chl content in *V. radiata* seedlings. However, 1 and 2 ppm of HZRP enhanced Chl content by 35 and 45%, respectively. In the case of *V. radiata* seedlings grown in Cr-toxic conditions, HZRP (2 ppm) enhanced Chl content by more than onefold in comparison with Cr-only treatment. Contrarily, Cr stress significantly increased the Chlase activity in *V. radiata* seedlings. Both applied concentrations of HZRP decreased the Chlase activity in *V. radiata* seedlings grown in Cr-contaminated soil.

### Harzianopyridone Mitigated Chromium Stress-Induced Oxidative Damage in *Vigna radiata*

**FIGURE 2:**
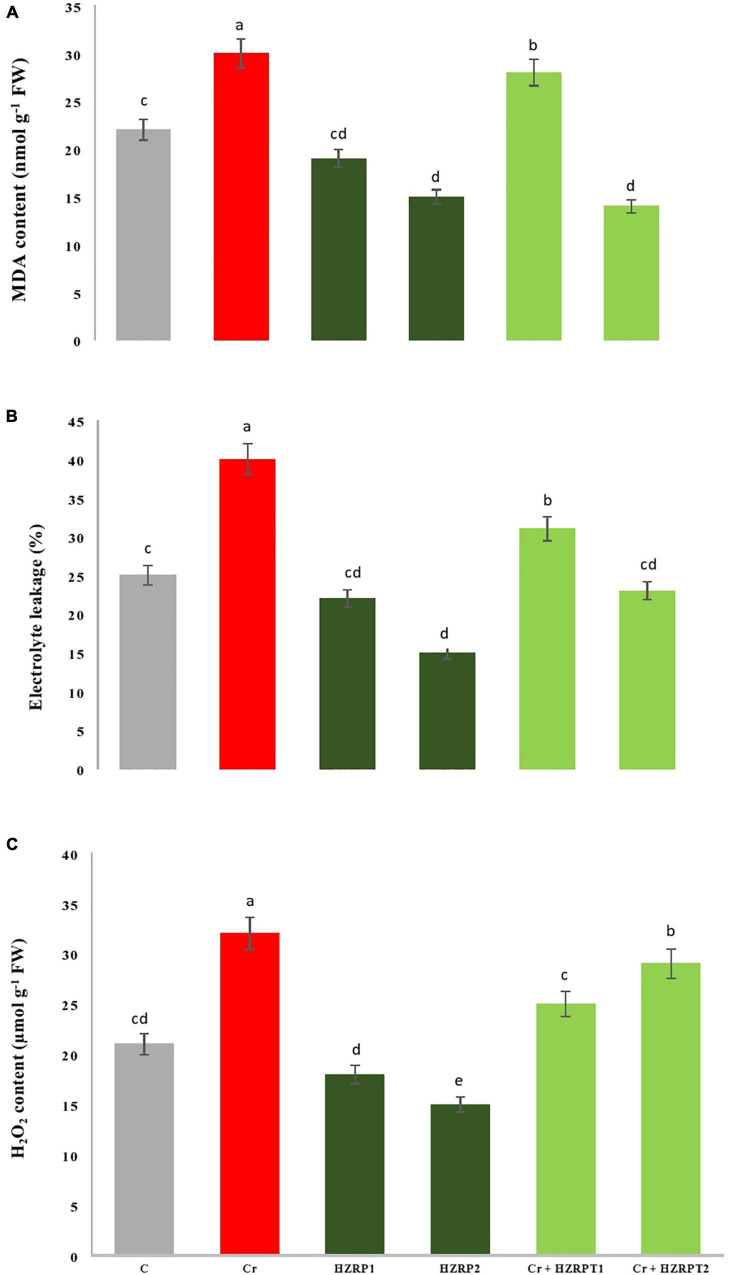
Effect of HZRP on malondialdehyde content **(A)**, electrolyte leakage **(B)** and hydrogen peroxide content **(C)** in *V. radiata* seedlings grown in Cr toxificaled soil. Different letters indicate significant difference among the treatments (*p* < 0.05). Cr, 50 mg kg^–1^, HZRP1, 1 p.p.m; HZRP2, 2 p.p.m.

Chromium can induce oxidative stress on the plant by the generation of ROS. Consequently, the destruction of membrane lipids under cobalt stress could increase the MDA content and EL. Here, Cr toxicity increased MDA content, EL, and H_2_O_2_ content by 36, 60, and 52%, respectively, as compared with control-treated *V. radiata* seedlings. Interestingly, HZRP supplementation reduced MDA content and EL in *V. radiata* seedlings grown in normal and Cr-exposed soil. A high concentration of HZRP (2 ppm) significantly reduced MDA content in *V. radiata* as compared with Cr-only treatment. In the case of *V. radiata* seedlings grown in Cr-toxificated soil, HZRP (2 ppm) treatment reduced MDA content by > 1-fold in comparison with Cr-only treatment. HZRP (2 ppm) treatment also reduced EL and H_2_O_2_ content in *V. radiata* seedlings grown in Cr-polluted soil (Figure 2).

### Harzianopyridone Improved the Redox Status of Chromium Stressed *Vigna radiata* Seedlings

**FIGURE 3:**
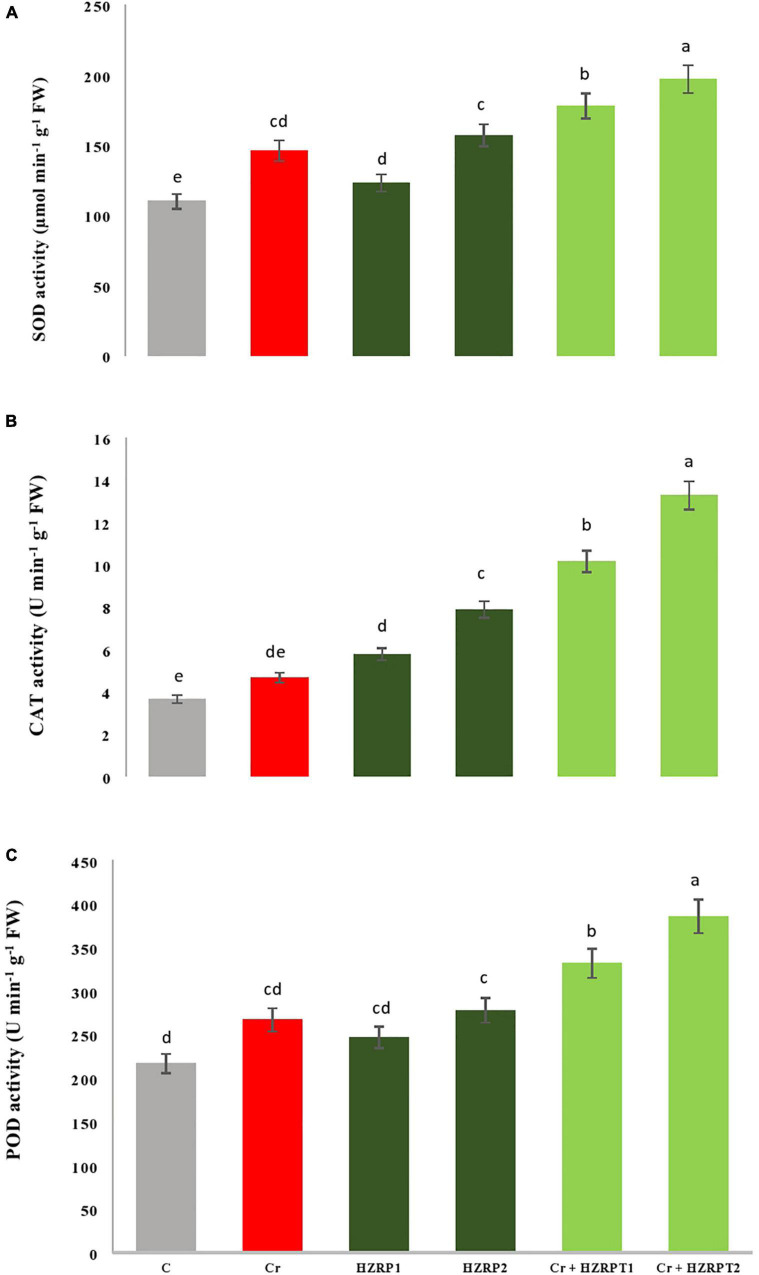
Effect of HZRP on SOD **(A)**, CAT **(B)** and POD **(C)** activity in *V. radiata* seedlings grown in Cr toxificaled soil. Different letters indicate significant difference among the treatments (*p* < 0.05). Cr. 50 mg kg^1^, HZRP1, 1 p.p.m; HZRP2. 2 p.p.m.

To cope with stress conditions, plants might induce antioxidants, which could play a role in mitigating the detrimental effects of heavy metal stress. In addition, HZRP might contribute to increasing the antioxidant metabolites levels and antioxidant enzyme activities to reduce the oxidative stress under Cr stress. Our results indicated that Cr stress escalated the activity of antioxidant enzymes (SOD, CAT, and POD) in *V. radiata* seedlings. The two priming concentrations of HZRP (1 and 2 ppm) significantly increased SOD, CAT, and POD activities in *V. radiata* seedlings grown in Cr-toxificated soil, in comparison to the Cr-only treatment. In the case of normal soil, priming of 2 ppm HZRP increases SOD activity by 42% in *V. radiata* seedlings grown in normal soil. It also increased SOD activity by 34% in *V. radiata* seedlings grown in Cr-polluted soil. Both the two priming concentrations of HZRP (1 and 2 ppm) also escalated POD activity in *V. radiata* seedlings grown in normal and Cr-contaminated potted soil. In the case of *V. radiata* seedlings grown in Cr-toxificated soil, HZRP increased the POD activity by 44% in comparison with the Cr-only treatment. Furthermore, they increased the activity of the CAT enzyme in *V. radiata* seedlings grown in normal and Cr-polluted potted (Figure 3).

**FIGURE 4:**
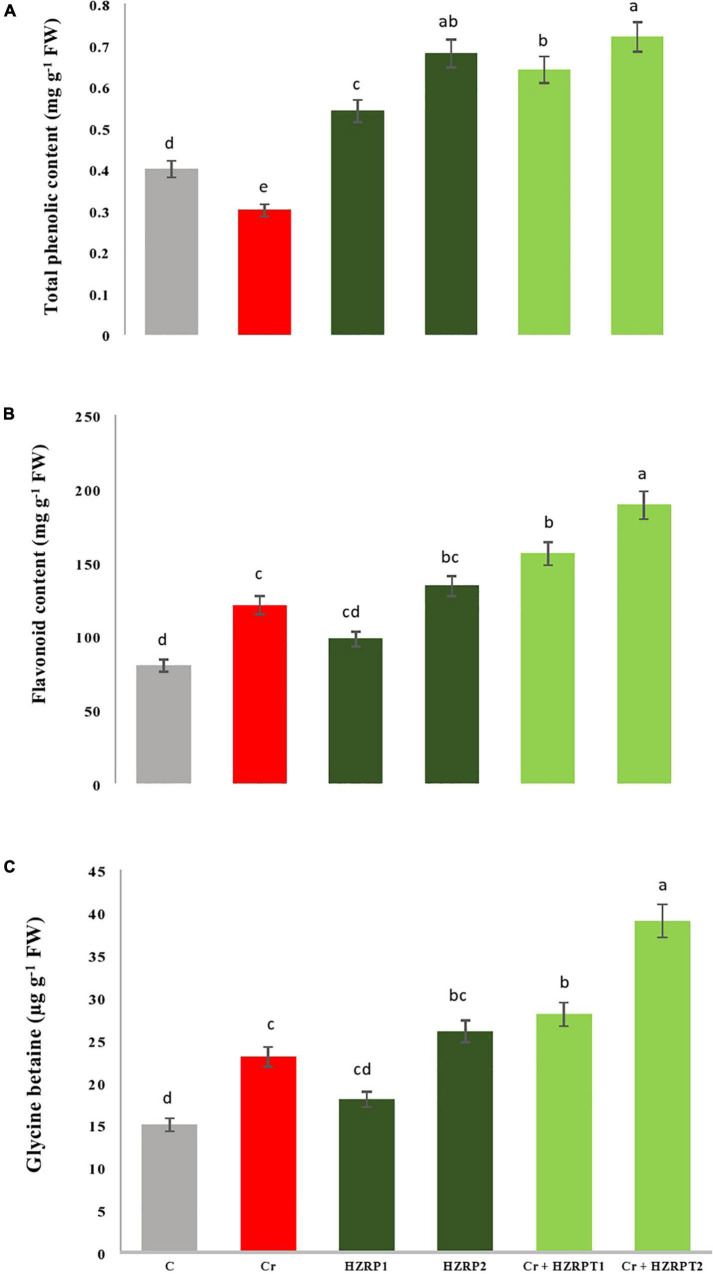
Effect of HZRP on total phenolic content **(A)**, flavonoids **(B)** and glycine betaine **(C)** content in *V. radiata* seedlings grown in *CT* toxificated soil. Different letters indicate significant difference among the treatments (*p* < 0.05). Cr, 50 mgkg^–1^, H7.RP1, 1 p.p.m; HZRP2. 2 p.p.m.

At the antioxidant metabolic level, Cr stress decreased total phenolic content (33%) in *V. radiata* seedlings as compared to C-treatment. Contrarily, Cr stress enhanced flavonoid content by 51% as compared to *V. radiata* seedlings grown in the control treatment. Priming with 2 ppm of HZRP significantly enhanced total phenolic content in normal and Cr-contaminated soil as compared with C-treatment. It also increased flavonoid content by 56% in *V. radiata* seedlings grown in Cr-polluted potted soil. Similarly, priming with both concentrations of HZRP significantly increased glycine betaine content in *V. radiata* seedlings grown in normal and Cr-polluted potted soil (Figure 4).

## Discussion

Globally, Cr pollution in the environment is one of the key reasons for the deterioration in ecosystem sustenance. Cr is one of the toxic heavy metals with hazardous effects on plants and human health. During this study, the effect of Cr on growth and physicochemical parameters was also investigated. Amin et al. (2013) reported that Cr stress reduced seed germination in *Hibiscus esculentus* and other legume crops.

**FIGURE 5:**
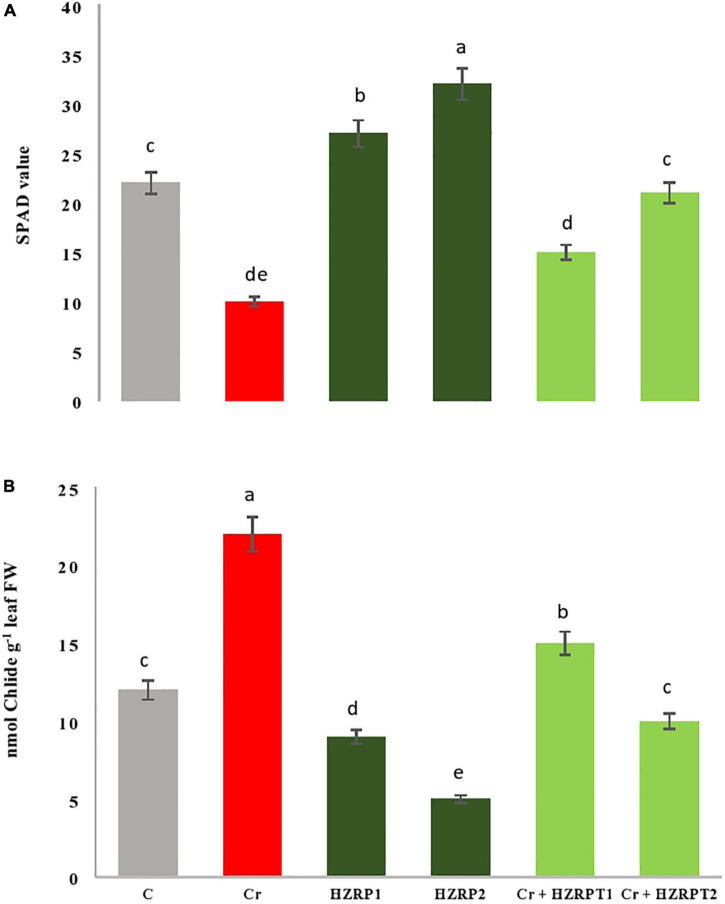
Effect of harzianopyridone on Ch1 content **(A)** and chlorophyllase activity **(B)** in *Vigna radiata* seedlings grown in Cr toxificated soil. Different letters indicate significant difference among the treatments (*p* < 0.05). Cr, 50 mg kg^–1^, HZRP1, 1 p.p.m; HZRP2, 2 p.p.m.

Chromium toxicity reduced growth, net photosynthetic rate, and gas exchange attributes in *V. radiata* seedlings. Alamri et al. (2020) also reported an increase in the activation of Chl degrading enzyme Chlase in tomato seedlings exposed to hexavalent Cr stress. During this study, Figure 5 shows that Cr toxicity enhanced Chlase activity, which reduced Chl content, leading to a decrease in net photosynthetic rate and photosynthate production in *V. radiata* seedlings.

Increased levels of MDA, EL, and H_2_O_2_ advocated oxidative stress in mung beans. Increased content of these oxidative stress markers disturbed the equilibrium between the antioxidative defensive approach and ROS accumulation. Similar results are reported in *Zea mays* (Anjum et al., 2017), *Brassica napus* (Gill et al., 2016), and *Cicer arietinum* (Singh D. et al., 2020).

Accumulation of H_2_O_2_ in plants is a crucial stress marker that results in oxidative stress in plants (Sharma et al., 2012). At higher concentrations, H_2_O_2_ disturbs the crucial physiological processes in plants, such as photosynthesis, respiration, stomatal conductance, and cellular development (Noctor et al., 2002). Current research showed an increase in H_2_O_2_ content in *V. radiata* seedlings exposed to Cr stress. This increase in H_2_O_2_ content resulted in a disturbance in normal physiological processes in *V. radiata* seedlings. Contrarily, HZRP treated seedlings showed reduced H_2_O_2_ levels.

Figure 3 shows that HZRP treatment enhanced the activities of SOD, CAT, and POD in *V. radiata* seedlings in normal and Cr-toxic conditions. Exogenous application of 2 ppm HZRP significantly increased the activity of the antioxidant enzyme, which reduced MDA, EL, and H_2_O_2_ content in treated *V. radiata* seedlings. SOD is an important line of defense in plants against stresses. SOD converts O^⋅⁣–^_2_ into O_2_ and H_2_O_2_. This conversion reduces OH^⋅^ formation. The activity of SOD is found to be upregulated in plants facing stressed conditions (Das and Roychoudhury, 2014). Current research reveals that HZRP application increased the activity of antioxidative enzymes (SOD, CAT, and POD) in *V. radiata* seedlings exposed to Cr stress.

Flavonoids are crucial for stress responses in plants. Flavonoids are involved in the scavenging of ROS produced in plants exposed to stress (Di Ferdinando et al., 2012). These secondary metabolites are involved in the stabilization of photosynthetic apparatus in plants (Stefanov et al., 2021). Glycine betaine is a crucial organic osmolyte that plays a pivotal role in mediating osmotic balance in plants facing stressed conditions (Ashraf and Foolad, 2007). This study showed HZRP-treated seedlings showed an increase in flavonoid content, which might have reduced ROS, thereby leading to Cr stress alleviation in *V. radiata* seedlings exposed to Cr stress.

Glycine betaine is reported to alleviate numerous abiotic stresses in sorghum (Kumar, 2021). The present study revealed that HZRP treatment alleviated Cr toxicity in *V. radiata* seedlings. This alleviation in Cr stress might be due to a reduction in Cr uptake in treated seedlings. It means that MDA and oxidative stress markers were not increased, which maintained Chl content, net photosynthetic rate, stomatal conductance, and intercellular CO_2_ concentration in *V. radiata* seedlings exposed to Cr stress. Aamer et al. (2018) reported that foliar application of glycine betaine alleviated Cd stress in *Spinacia oleraceae* through a reduction in Cd uptake and increased the activity of the antioxidative defensive system.

The plant has developed various adaptations to detoxify Cr content, such as Cr uptake (Shahid et al., 2017). Similar results have been reported in *Oryza sativa* (Chen et al., 2017), *Arabidopsis thaliana* (Wakeel et al., 2019), and *Brassica juncea* (Handa et al., 2018). Current studies depicted that HZRP supplementation reduced Cr uptake in *V. radiata* seedlings. This reduced Cr uptake regulated the growth and physiochemical properties of treated seedlings.

## Conclusion

**FIGURE 6:**
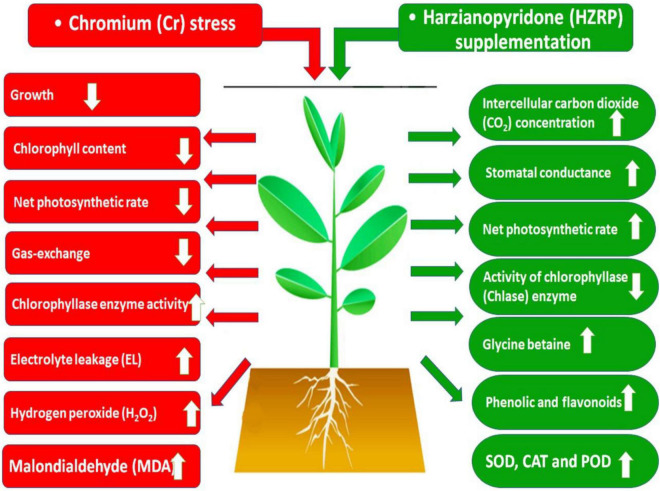
Schematic model explaining the role of HZRP in alleviation of Cr stress in *V. radiata* seedlings.

Chromium stress reduced the growth of *V. radiata* seedlings in potting soil. Cr stress decreased Chl content, net phostosynthetic rate, besides an increase in Chlase activity. Increased levels of EL, MDA, and H_2_O_2_ were observed in *V. radiata* seedlings exposed to Cr-toxificated soil. Contrarily, HZRP increased the activities of SOD, CAT, and POD in *V. radiata* seedlings. Apart from this, HZRP increased total phenolic content, flavonoids, and glycine betaine level in treated seedlings. Moreover, HZRP supplementation reduced Cr uptake in *V. radiata* exposed seedlings exposed to Cr stress. It is proposed that HZRP and other volatile organic compounds may be exploited for abiotic stress tolerance in plants (Figure 6).

## Data Availability Statement

The original contributions presented in this study are included in the article/supplementary material, further inquiries can be directed to the corresponding author.

## Author Contributions

AAS and ANS: experimentation and validation. MB: research design. AA and SJ: statistical analysis. MR: validation. SA, HA, and TJ: Research design and review and drafting. SSA, AT, and HMK: Funding acquisition and review and drafting. All authors contributed to the article and approved the submitted version.

## Conflict of Interest

The authors declare that the research was conducted in the absence of any commercial or financial relationships that could be construed as a potential conflict of interest.

## Publisher’s Note

All claims expressed in this article are solely those of the authors and do not necessarily represent those of their affiliated organizations, or those of the publisher, the editors and the reviewers. Any product that may be evaluated in this article, or claim that may be made by its manufacturer, is not guaranteed or endorsed by the publisher.
